# Code of Medical Ethics

**Published:** 1864-07

**Authors:** 


### SELECTED.

[We publish the following at the reqnest of a number of the junior members of the profession among our subscribers. —Eds.]

### CODE OF MEDICAL ETHICS.

### OF THE DUTIES OF PHYSICIANS TO THEIR PATIENTS, AND OF THE OBLIGATIONS OF PATIENTS TO THEIR PHYSICIANS.

ART. I.—*Duties of Physicians to their Patients.*

§ 1. A physician should not only be ever ready to obey the calls of the sick, but his mind ought also to be imbued with the greatness of his mission, and the responsibility he habitually incurs in its discharge. Those obligations are the more deep and enduring, because there is no tribunal other than his own conscience to adjudge penalties for carelessness or neglect. Physicians should, therefore, minister to the sick with due impressions of the importance of their office; reflecting that the ease, the health, and the lives of those committed to their charge, depend on their skill, attention, and fidelity. They should study, also, in their deportmert, so to unite *tenderness* with *firmness*, and *condescension* with *authority*, as to inspire the minds of their patients with gratitude, respect, and confidence.

§ 2. Every case committed to the charge of a physician should be treated with attention, steadiness, and humanity.

Reasonable indulgence should be granted to the mental imbecility and caprices of the sick. Secresy and delicacy, when required by peculiar circumstances, should be strictly observed; and the familiar and confidential intercourse to which physicians are admitted in their professional visits, should be used with discretion, and with the most scrupulous regard to fidelity and honor. The obligation of secresy extends beyond the period of professional services;—none of the privacies of personal and domestic life, no infirmity of disposition or flaw of character observed during professional attendance, should ever be divulged by the physician except when he is imperatively required to do so. The force and necessity of this obligation are indeed so great, that professional men have, under certain circumstances, been protected in their observance of secresy by courts of justice.

§ 3. Frequent visits to the sick are in general requisite, since they enable the physician to arrive at a more perfect knowledge of the disease—to meet promptly every change that may occur, and also tend to preserve the confidence of the patient. But unnecessary visits are to be avoided, as they give useless anxiety to the patient, tend to diminish the authority of the physician, and render him liable to be suspected of interested motives.

§ 4. A physician should not be forward to make gloomy prognostications, because they savor of empiricism, by magnifying the importance of his services in the treatment or cure of the disease. But he should not fail, on proper occasions, to give to the friends of the patient timely notice of danger when it really occurs; and even to the patient himself, if absolutely necessary. This office, however, is so peculiarly alarming when executed by him, that it ought to be declined whenever it can be assigned to any other person of sufficient judgment and delicacy. For the physician should be the minister of hope and comfort to the sick; that, by such cordials to the drooping spirit, he may smooth the bed of death, revive expiring life, and counteract the depressing influence of those maladies which often disturb the tranquility of the most resigned in their last moments. The life of a sick person can be shortened not only by the acts, but also by the words or the manner of a physician. It is, therefore, a sacred duty to guard himself carefully in this respect, and to avoid all things which have a tendency to discourage the patient, and to depress his spirits.

§ 5. A physician ought not to abandon a patient because the case is deemed incurable; for his attendance may continue to be highly useful to the patient, and comforting to the relatives around him, even in the last period of a fatal malady, by alleviating pain and other symptoms, and by soothing mental anguish. To decline attendance, under such circumstances, would be sacrificing to fanciful delicacy and mistaken liberality, that moral duty, which is independent of, and far superior to, all pecuniary consideration.

§ 6. Consultations should be promoted in difficult or protracted cases, as they give rise to confidence, energy, and more enlarged views in practice.

§ 7. The opportunity which a physician not unfrequently enjoys of promoting and strengthening the good resolutions of his patients, suffering under the consequences of vicious conduct, ought never to be neglected. His counsels, or even remonstrances, will give satisfaction, not offence, if they be proffered with politeness, and evince a genuine love of virtue, accompanied by a sincere interest in the welfare of the person to whom they are addressed.

ART. II.—*Obligations of Patients to their Physicians.*

§ 1. The members of the medical profession, upon whom is enjoined the performance of so many important and arduous duties towards the community, and who are required to make so many sacrifices of comfort, ease, and health, for the welfare of those who avail themselves of their services, certainly have a right to expect and require, that their patients should entertain a just sense of the duties which they owe to their medical attendants.

§ 2. The first duty of a patient is to select as his medical adviser one who has received a regular professional education. In no trade or occupation, do mankind rely on the skill of an untaught artist, and in medicine, confessedly the most difficult and intricate of the sciences, the world ought not to suppose that knowledge is intuitive.

§ 3. Patients should prefer a physician whose habits of life are regular, and who is not devoted to company, pleasure, or to any pursuit incompatible with his professional obligations. A patient should, also, confide the care of himself and family as much as possible, to one physician; for a medical man, who has become acquainted with the peculiarities of constitutions, habits, and predispositions, of those he attends, is more likely to be successful in his treatment than one who does not possess that knowledge.

A patient who has thus selected his physician, should always apply for advice in what may appear to him trivial cases, for the most fatal results often supervene on the slightest accidents. It is of still more importance that he should apply for assistance in the forming stage of violent diseases; it is to a neglect of this precept that medicine owes much of the uncertainty and imperfection with which it has been reproached.

§ 4. Patients should faithfully and unreservedly communicate to their physician the supposed cause of their disease. This is the more important, as many diseases of a mental origin simulate those depending on external causes, and yet are only to be cured by ministering to the mind diseased. A patient should never be afraid of thus making his physician his friend and adviser; he should always bear in mind that a medical man is under the strongest obligations of secresy. Even the female sex should never allow feelings of shame or delicacy to prevent their disclosing the seat, symptoms, and causes of complaints peculiar to them. However commendable a modest reserve may be in the common occurrences of life, its strict observance in medicine is often attended with the most serious consequences, and a patient may sink under a painful and loathsome disease, which might have been readily prevented had timely intimation been given to the physician.

§ 5. A patient should never weary his physician with a tedious detail of events or matters not appertaining to his disease. Even as relates to his actual symptoms, he will convey much more real information by giving clear answers to interrogatories, than by the most minute account of his own framing. Neither should he obtrude upon his physician the details of his business, nor the history of his family concerns.

§ 6. The obedience of a patient to the prescriptions of his physician should be prompt and implicit. He should never permit his own crude opinions as to their fitness, to influence his attention to them. A failure in one particular may render an otherwise judicious treatment dangerous, and even fatal. This remark is equally applicable to diet, drink, and exercise. As patients become convalescent, they are very apt to suppose that the rules prescribed for them may be disregarded, and the consequence, but too often, is a relapse. Patients should never allow themselves to be persuaded to take any medicine whatever, that may be recommended to them by the self-constituted doctors and doctresses, who are so frequently met with, and who pretend to possess infallible remedies for the cure of every disease. However simple some of their prescriptions may appear to be, it often happens that they are productive of much mischief, and in all cases they are injurious, by contravening the plan of treatment adopted by the physician.

§ 7. A patient should, if possible, avoid even the *friendly visits of a physician* who is not attending him—and when he does receive them, he should never converse on the subject of his disease, as an observation may be made, without any intention of interference, which may destroy his confidence in the course he is pursuing, and induce him to neglect the directions prescribed to him. A patient should never send for a consulting physician without the express consent of his own medical attendant. It is of great importance that physicians should act in concert; for, although their modes of treatment may be attended with equal success when employed singly, yet conjointly they are very likely to be productive of disastrous results.

§ 8. When a patient wishes to dismiss his physician, justice and common courtesy require that he should declare his reasons for so doing.

§ 9. Patients should always, when practicable, send for the physician in the morning, before his usual hour of going out; for, by being early aware of the visits he has to pay during the day, the physician is able to apportion his time in such a manner as to prevent an interference of engagements. Patients should also avoid calling on their medical adviser unnecessarily during the hours devoted to meals or sleep. They should always be in readiness to receive the visits of their physician, as the detention of a few minutes is often of serious inconvenience to him.

§ 10. A patient should, after his recovery, entertain a just and enduring sense of the value of the services rendered him by his physician; for these are of such a character, that no mere pecuniary acknowledgment can repay or cancel them.

---

### OF THE DUTIES OF PHYSICIANS TO EACH OTHER, AND TO THE PROFESSION AT LARGE.

### ART. I.—*Duties for the Support of Professional Character.*

§ 1. Every individual, on entering the profession, as he becomes thereby entitled to all its privileges and immunities, incurs an obligation to exert his best abilities to maintain its dignity and honor, to exalt its standing, and to extend the bounds of its usefulness. He should, therefore, observe strictly such laws as are instituted for the government of its members; —should avoid all contumelious and sarcastic remarks relative to the faculty, as a body; and while, by unwearied diligence, he resorts to every honorable means of enriching the science, he should entertain a due respect for his seniors, who have, by their labors, brought it to the elevated condition in which he finds it.

§ 2. There is no profession, from the members of which greater purity of character, and a higher standard of moral excellence are required, than the medical; and to attain such eminence, is a duty every physician owes alike to his profession and to his patients. It is due to the latter, as without it he can not command their respect and confidence, and to both because no scientific attainments can compensate for the want of correct moral principles. It is also incumbent upon the faculty to be temperate in all things, for the practice of physic requires the unremitting exercise of a clear and vigorous understanding; and, on emergencies, for which no professional man should be unprepared, a steady hand, an acute eye, and an unclouded head may be essential to the well-being, and even to the life, of a fellow-creature.

§ 3. It is derogatory to the dignity of the profession to resort to public advertisements, or private cards, or handbills, inviting the attention of individuals affected with particular diseases--publicly offering advice and medicine to the poor gratis, or promising radical cures; or to publish cases and operations in the daily prints, or suffer such publications to be made; to invite laymen to be present at operations; to boast of cures and remedies; to adduce certificates of skill and success, or to perform any other similar acts. These are the ordinary practices of empirics, and are highly reprehensible in a regular physician.

§ 4. Equally derogatory to professional character is it, for a physician to hold a patent for any surgical instrument or medicine; or to dispense a secret *nostrum*, whether it be the composition or exclusive property of himself or of others. For, if such nostrum be of real efficacy, any concealment regarding it is inconsistent with beneficence and professional liberality; and, if mystery alone give it value and importance such craft implies either disgraceful ignorance or fraudulent avarice. It is also teprehensible for physicians to give certificates attesting the efficacy of patent or secret medicines, or in any way to promote the use of them.

ART. II.—*Professional Services of Physicians to each other.*

§ 1. All practitioners of medicine, their wives, and their children while under the paternal care, are entitled to the gratuitous services of any one or more of the faculty residing near them, whose assistance may be desired. A physician afflicted with disease is usually an incompetent judge of his own case; and the natural anxiety an solicitude which he experiences at the sickness of a wife, a child, or any one who, by the ties of consanguinity, is rendered peculiarly dear to him, tend to obscure his judgment, and produce timidity and irresolution in his practice. Under such circumstances, medical men are peculiarly dependent upon each other, and kind offices and professional aid should always be cheerfully and gratuitously afforded. Visits ought not, however, to be obtruded officiously; as such unasked civility may give rise to embarrassment, or interfere with that choice on which confidence depends. But, if a distant member of the faculty, whose circumstances are affluent, request attendance, and an honorarium be offered, it should not be declined, for no pecuniary obligation ought to be imposed, which the party receiving it would wish not to incur.

ART. III.—*Of the duties of Physicians as respects vicarious offices.*

§ 1. The affairs of life, the pursuit of health, and the various accidents and contingencies to which a medical man is peculiarly exposed, sometimes require him temporarily to withdraw from his duties to his patients, and to request some of his professional brethren to officiate for him. Compliance with this request is an act of courtesy, which should always be performed with the utmost consideration for the interest and character of the family physician, and when exercised for a short period, all the pecuniary obligations for such service should be awarded to him. But if a member of the profession neglect his business in quest of pleasure and amusement, he can not be considered as entitled to the advantages of the frequent and long-continued exercise of this fraternal courtesy without awarding to the physician who officiates the fees arising from the discharge of his professional duties.

In obstetrical and important surgical cases, which give rise to unusual fatigue, anxiety, and responsibility, it is just that the fees accruing therefrom should be awarded to the physician who officiates.

ART. IV.—*Of the duties of Physicians as regards Consultations.*

§ 1. A regular medical education furnishes the only presumptive evidence of professional abilities and acquirements, and ought to be the only acknowledged right of an individual to the exercise and honors of his profession. Nevertheless, as in consultations the good of the patient is the sole object in view, and this is often dependent on personal confidence, no intelligent regular practitioner, who has a license to practice from some medical board of known and acknowledged respectability, recognized by this association, and who is in good moral and professional standing in the place in which he resides, should be fastidiously excluded from fellowship, or his aid refused in consultation, when it is requested by the patient. But no one can be considered as a regular practitioner or a fit associate in consultation, whose practice is based on an exclusive dogma, to the rejection of the accumulated experience of the profession, and of the aids actually furnished by anatomy, physiology, pathology, and organic chemistry.

§ 2. In consultations, no rivalship or jealousy should be indulged; candor, probity, and all due respect should be exercised towards the physician having charge of the case.

§ 3. In consultations, the attending physician should be the first to propose the necessary questions to the sick; after which the consulting physician should have the opportunity to make such further inquiries of the patient as may be necessary to satisfy him of the true character of the case. Both physicians should then retire to a private place for deliberation; and the one first in attendance should communicate the directions agreed upon to the patient or his friends, as well as any opinions which it may be thought proper to express. But no statement or discussion of it should take place before the patient or his friends, except in the presence of all the faculty attending, and by their common consent; and no *opinions* or *prognostications* should be delivered, which are not the result of previous deliberation and concurrence.

§ 4. In consultations, the physician in attendance should deliver his opinion first; and when there are several consulting, they should deliver their opinions in the order in which they have been called in. No decision, however, should restrain the attending physician from making such variations in the mode of treatment, as any subsequent unexpected change in the character of the case may demand. But such variation and the reasons for it, ought to be carefully detailed at the next meeting in consultation. The same privilege belongs also to the consulting physician if he is sent for in an emergency, when the regular attendant is out of the way, and similar explanations must be made at the next consultation.

§ 5. The utmost punctuality should be observed in the visits of physicians when they are to hold consultation together, and this is generally practicable, for society has been considerate enough to allow the plea of a professional engagement to take precedence of all others, and to be an ample reason for the relinquishment of any present occupation. But as professional engagements may sometimes interfere, and delay one of the parties, the physician who first arrives should wait for his associate a reasonable period, after which the consultation should be considered as postponed to a new appointment. If it be the attending physician who is present, he will of course see the patient and prescribe; but if it be the consulting one, he should retire, except in case of emergency, or when he has been called from a considerable distance, in which latter case he may examine the patient, and give his opinion *in writing*, and *under seal*, to be delivered to his associate.

§ 6. In consultations, theoretical discussions should be avoided, as occasioning perplexity and loss of time. For there may be much diversity of opinion concerning speculative points, with perfect agreement in those modes of practice which are founded, not on hypothesis, but on experience and observation.

§ 7. All discussions in consultation should be held as secret and confidential. Neither by words or manner should any of the parties to a consultation assert or insinuate, that any part of the treatment pursued did not receive his assent. The responsibility must be equally divided between the medical attendants—they must equally share the credit of success as well as the blame of failure.

§ 8. Should an irreconcilable diversity of opinion occur when several physicians are called upon to consult together, the opinion of the majority should be considered as decisive; but if the numbers be equal on each side, then the decision should rest with the attending physician. It may, moreover, sometimes happen, that two physicians can not agree in their views of the nature of a case, and the treatment to be pursued. This is a circumstance much to be deplored, and should always be avoided, if possible, by mutual concessions, as far as they can be justified by a conscientious regard for the dictates of judgment. But, in the event of occurrence, a third physician should, if practicable, be called to act as umpire; and, if circumstance prevent the adoption of this course, it must be left to the patient to select the physician in whom he is most willing to confine. But, as every physician relies upon the rectitude of his judgment, he should, when left in the minority, politely and consistently retire from any farther deliberation in the consultation, or participation in the management of the case.

§ 9. As circumstances sometimes occur to render a *special consultation* desirable when the continued attendance of two physicians might be objectionable to the patient, the member of the faculty whose assistance is required in such cases, should sedulously guard against all future unsolicited attendance. As such consultations require an extraordinary portion both of time and attention, at least a double honorarium may reasonably be expected.

§ 10. A physician who is called upon to consult, should observe the most honorable and scrupulous regard for the character and standing of the practitioner in attendance; the practice of the latter, if necessary, should be justified as far as it can be, consistently with a conscientious regard for truth, and no hint or insinuation should be thrown out which could impair the confidence reposed in him, or affect his reputation. The consulting physician should also carefully refrain from any of those extraordinary attentions or assiduities, which are too often practiced by the dishonest for the base purpose of gaining applause, or ingratiating themselves into the favor of families and individuals.

ART. V.—*Duties of Physicians in cases of Interference.*

§ 1. Medicine is a liberal profession, and those admitted into its ranks should found their expectations of practice upon the extent of their qualifications, not on intrigue or artifice.

§ 2. A physician in his intercourse with a patient under the care of another practitioner, should observe the strictest reserve and caution. No meddling inquiries should be made—no disingenuous hints given relative to the nature and treatment of his disorder; nor any course of conduct pursued that may directly or indirectly tend to diminish the trust reposed in the physician employed.

§ 3. The same circumspection and reserve should be observed when, from motives of business or friendship, a physician is prompted to visit an individual who is under the direction of another practitioner. Indeed, such visits should be avoided, except under peculiar circumstances; and when they are made, no particular inquiries should be instituted relative to the nature of the disease, or the remedies employed, but the topic of conversation should be as foreign to the case as circumstances will admit.

§ 4. A physician ought not to take charge of or prescribe for a patient who has recently been under the care of another member of the faculty in the same illness, except in cases of sudden emergency, or in consultation with the physician previously in attendance, or when the latter has relinquished the case, or been regularly notified that his services are no longer desired. Under such circumstances, no unjust and illiberal insinuations should be thrown out in relation to the conduct or practice previously pursued, which should be justified as far as candor and regard for truth and probity will permit; for it often happens that patients become dissatisfied when they do not experience immediate relief, and, as many diseases are naturally protracted, the want of success, in the first stage of treatment, affords no evidence of a lack of professional knowledge and skill.

§ 5. When a physician is called to an urgent case, because the family attendant is not at hand, he ought, unless his assistance in consultation be desired, to resign the care of the patient to the latter immediately on his arrival.

§ 6. It often happens, in cases of sudden illness, or of recent accidents and injuries, owing to the alarm and anxiety of friends, that a number of physicians are simultaneously sent for. Under these circumstances, courtesy should assign the patient to the first who arrives, who should select from those present, any additional assistance that he may deem necessary. In all such cases, however, the practitioner who officiates should request the family physician, if there be one, to be called, and, unless his farther attendance be requested, should resign the case to the latter on his arrival.

§ 7. When a physician is called to the patient of another practitioner, in consequence of the sickness or absence of the latter, he ought, on the return or recovery of the regular attendant, and with the consent of the patient, to surrender the case.

[The expression, "Patient of another Practitioner," is understood to mean a patient who may have been under the charge of another practitioner at the time of the attack of sickness, or departure from home, of the latter, or who may have called for his attendance during his absence or sickness, or in any other manner given it to be understood that he regarded the said physician as his regular medical attendant.]

§ 8. A physician when visiting a sick person in the country may be desired to see a neighboring patient who is under the regular direction of another physician, in consequence of some sudden change or aggravation of symptoms. The conduct to be pursued on such an occasion is to give advice adapted to present circumstances; to interfere no further than is absolutely necessary with the general plan of treatment; to assume no future direction, unless it be expressly desired; and, in this last case, to request an immediate consultation with the practitioner previously employed.

§ 9. A wealthy physician should not give advice *gratis* to the affluent; because his doing so is an injury to his professional brethren. The office of a physician can never be supported as an exclusively beneficent one; and it is defrauding, in some degree, the common funds for its support, when fees are dispensed which might justly be claimed.

§ 10. When a physician who has been engaged to attend a case of midwifery is absent, and another is sent for, if delivery is accomplished during the attendance of the latter, he is entitled to the fee, but should resign the patient to the practitioner first engaged.

Art. VI.—*Of Differences between Physicians.*

§ 1. Diversity of opinion and opposition of interest, may, in the medical as in other professions, sometimes occasion controversy and even contention. Whenever such cases unfortunately occur, and can not be immediately terminated, they should be referred to the arbitration of a sufficient number of physicians, or a *court-medical.*

§ 2. As peculiar reserve must be maintained by physicians towards the public, in regard to professional matters, and as there exist numerous points in medical ethics and etiquette through which the feelings of medical men may be painfully assailed in their intercourse with each other, and which can not be understood or appreciated by general society, neither the subject-matter of such differences nor the adjudication of the arbitrators should be made public, as publicity in a case of this nature may be personally injurious to the individuals concerned, and can hardly fail to bring discredit on the faculty.

ART. VII.—*Of Pecuniary Acknowledgments.*

Some general rules should be adopted by the faculty, in every town or district, relative to *pecuniary acknowledgments* from their patients; and it should be deemed a point of honor to adhere to these rules with as much uniformity as varying circumstances will admit.

---

### OF THE DUTIES OF THE PROFESSION TO THE PUBLIC, AND OF THE OBLIGATIONS OF THE PUBLIC TO THE PROFESSION.

ART. I.—*Duties of the Profession to the Public.*

§ 1. As good citizens, it is the duty of physicians to be ever vigilant for the welfare of the community, and to bear their part in sustaining its institutions and burdens; they should also be ever ready to give counsel to the public in relation to matters especially appertaining to their profession, as on subjects of medical police, public hygiene, and legal medicine. It is their province to enlighten the public in regard to quarantine regulations—the location, arrangement, and dietaries of hospitals, asylums, schools, prisons, and similar institutions—in relation to the medical police of towns, as drainage, ventilation, etc.—and in regard to measures for the prevention of epidemic and contagious diseases; and when p stilence prevails, it is their duty to face the danger, and to continue their labors for the alleviation of the suffering, even at the jeopardy of their own lives.

§ 2. Medical men should be always ready, when called on by the legally constituted authorities, to enlighten coroners' inquests, and courts of justice, on subjects strictly medical—such as involve questions relating to sanity, legitimacy, murder by poisons or other violent means, and in regard to the various other subjects embraced in the science of Medical Jurisprudence. But in these cases, and especially where they are required to make *post-mortem* examination, it is just, in consequence of the time, labor, and skill required, and the responsibility and risk they incur, that the public should award them a proper honorarium.

§ 3. There is no profession, by the members of which eleemosynary services are more liberally dispensed than the medical, but justice requires that some limit should be placed to the performance of such good offices. Poverty, professional brotherhood, and certain of the public duties referred to in the first section of this article, should always be recognized as presenting valid claims for gratuitous services; but neither institutions endowed by the public or by rich individuals, societies for mutual benefit, for the insurance of lives, or for analogous purposes, nor any profession or occupation, can be admitted to possess such privilege. Nor can it be justly expected of physicians to furnish certificates of inability to serve on juries, to perform military duty, or to testify to the state of health of persons wishing to insure their lives, obtain pensions or the like, without a pecuniary acknowledgment. But to individuals in indigent circumstances, such professional services should always be cheerfully and freely accorded.

§ 4. It is the duty of physicians, who are frequent witnesses of the enormities committed by quackery, and the injury to health and even destruction of life caused by the use of quack medicines, to enlighten the public on these subjects, to expose the injuries sustained by the unwary from the devices and pretensions of artful empirics and imposters. Physicians ought to use all the influence which they may possess, as professors in Colleges of Pharmacy, and by exercising their option in regard to the shops to which their prescriptions shall be sent, to discourage druggists and apothecaries from vending quack or secret medicines, or from being in any way engaged in their manufacture and sale.

### ART. II.—*Obligations of the Public to Physicians.*

§ 1. The benefits accruing to the public, directly and indirectly, from the active and unwearied beneficence of the profession, are so numerous and important, that physicians are justly entitled to the utmost consideration and respect from the community. The public ought likewise to entertain a just appreciation of medical qualifications; to make a proper discrimination between true science and the assumptions of ignorance and empiricism—to afford every encouragement and facility for the acquisition of medical education—and no longer to allow the statute books to exhibit the anomaly of exacting knowledge from physicians, under a liability to heavy penalties, and of making them obnoxious to punishment for resorting to the only means of obtaining it.

